# Comparison of Pentaspline Pulsed Field Ablation Catheter Mapping Modes to Assess Pulmonary Vein Isolation for Paroxysmal Atrial Fibrillation

**DOI:** 10.1111/jce.70408

**Published:** 2026-07-19

**Authors:** John L. Fitzgerald, Geoffroy Ditac, Konstantinos Vlachos, Nicolas Johner, Xavier Paul Bouteiller, Kinan Kneizeh, Francesco Notaristefano, Karim Benali, Cinzia Monaco, Marianne T. Langlois, Benjamin Sacristan, Jan Charton, Romain Tixier, Nicolas Derval, Thomas Pambrun, Mélèze Hocini, Michel Haïssaguerre, Josselin Duchateau, Pierre Jaïs, Frédéric Sacher

**Affiliations:** ^1^ Department of Cardiac Pacing and Electrophysiology, Hôpital Cardiologique du Haut‐Leveque Bordeaux University Hospital Pessac France; ^2^ IHU Liryc, Electrophysiology and Heart Modeling Institute, INSERM 1045 University of Bordeaux Pessac France; ^3^ Centre for Heart Rhythm Disorders University of Adelaide Adelaide Australia; ^4^ Department of Electrophysiology, Hôpital Cardiologique Louis Pradel, Hospices Civils de Lyon Lyon France; ^5^ Cardiology Department American Hospital of Paris Paris France

**Keywords:** electro‐anatomic mapping, pulmonary vein isolation, pulsed field ablation

## Abstract

**Background:**

We assessed the electro‐anatomic mapping (EAM) capabilities of a pulsed field ablation (PFA) system previously guided by fluoroscopy to assess acquisition modes, and effects of near and far‐field signal.

**Methods:**

After pulmonary vein isolation (PVI) proven by pacing to check entrance and exit block, EAM was performed in 2 modes with the Faraview system during CS pacing. Mode 1 utilized bipoles between electrode 3 of each adjacent catheter spline. Mode 2 utilized a pseudo‐unipole between electrode 3 and a combination of electrodes 1, 2, and 4 on individual splines. Signals and voltage map demonstration of PVI were compared with pacing assessment. Two observers assessed the maps with a third observer to resolve discrepancies.

**Results:**

Fifteen patients (87% male, aged 58 ± 9 years) had PVI then mapping with the Faraview system. Pacing confirmed isolation of all pulmonary veins. Bipolar mapping (mode 1) displayed multiple apparent PV signals: only 32/61 (52%) of veins appeared isolated. On pseudo‐unipolar mapping 59/61 (97%) of veins appeared isolated. The majority of variance in assessment (70.4%) was explainable by the mapping method with minimal variance attributed to observer.

**Conclusions:**

Pseudo‐unipolar EAM Faraview mode appears the most accurate for assessment post‐PVI compared with bipolar mode.

## Graphical abstract

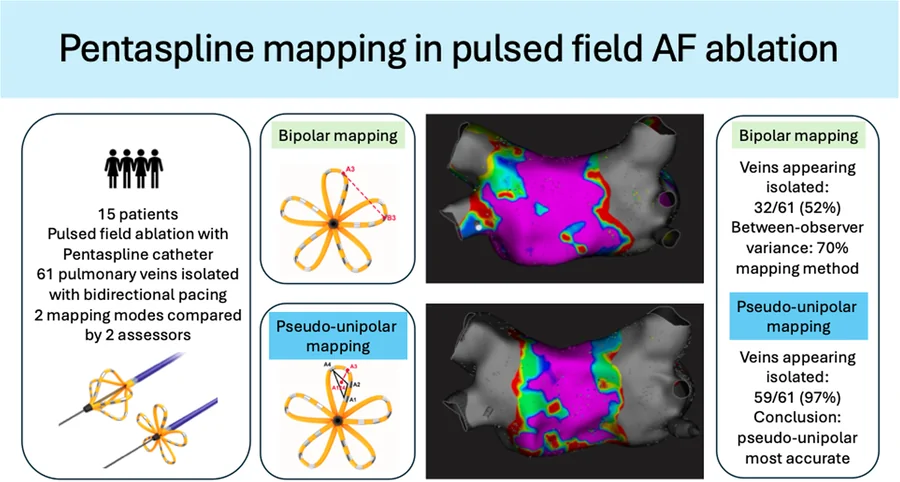

**Abbreviations**
3D EAM: 3‐Dimensional electro‐anatomic mapping
AF: atrial fibrillation
PFA: pulsed field ablation
RF: radiofrequency

## 1 Background

Pulsed field ablation (PFA) for atrial fibrillation (AF) has been widely adopted due to safety advantages, efficiency and at least equivalent efficacy to pre‐existing thermal technologies [1]. Farapulse, (Boston Scientific) a penta‐spline catheter with 2 treatment configurations known as “basket” and “flower” poses, was one of the first PFA systems to gain approval for widespread clinical use, and much of the clinical data to date involves this system [2]. Recent data shows that at repeat procedures for AF after pulsed field ablation with the pentaspline catheter, over half of patients are found to have pulmonary vein reconnection, though with reduced reconnection associated with mapping at the initial procedure [3]. This mapping involved the use of traditional 3‐dimensional electro‐anatomic mapping systems (EAM) available at the time, though Farapulse was initially developed to allow use with fluoroscopy guidance alone. This finding indicates there is clearly room for improvement in obtaining durable PVI.

**Figure 1:**
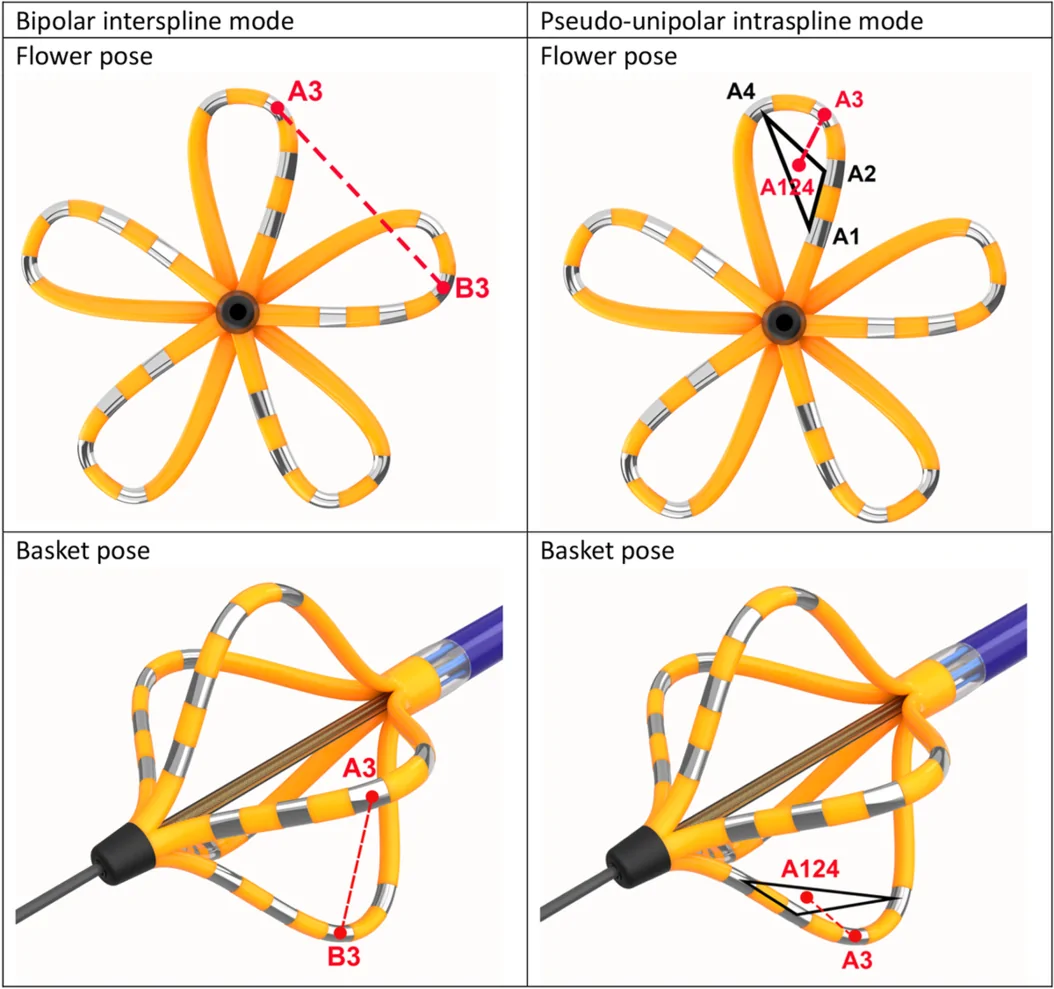
Catheter electrode recording configurations. Bipolar “interspline” mode records between electrode 3 on 2 adjacent splines. Pseudo‐unipolar “intraspline” mode records between electrode 3 and a virtual electrode comprised of a combination of electrodes 1,2, and 4 on the same spline. Here for illustrative purposes a single pseudo‐unipole and bipole are shown for each configuration, while all splines are actually involved in recording simultaneously. Electrode size is 8.8 mm^2^ and interspline configuration electrode spacing varies between 16 and 21 mm.

A recent development involves a new mapping option utilising the Farawave Nav ablation catheter with the The OPAL HDx mapping system (Boston Scientific, Natick, MA, USA) to generate 3D geometry and voltage or activation maps which can be used to guide ablation and to assess PVI post‐ablation. This mapping mode utilises the existing pentaspline catheter design, which was not developed for mapping purposes and has large electrodes and large inter‐electrode spacing. This raises challenges via known effects of increasing far‐field components of signals and reducing capacity to detect gaps between lesions compared to smaller, more closely‐spaced electrodes [4]. To overcome this problem, a virtual electrode configuration mode has been devised. Mapping can be performed in 2 modes: one with bipoles which are between electrode 3 on adjacent splines (“interspline”) with an electrode surface area of 8.8 mm^2^ and an inter‐electrode spacing of 16–21 mm, depending on the choice of 31 mm or 35 mm diameter catheter [5] and; the other utilising electrode 3 and a virtual electrode comprising electrodes 1, 2, and 4 of the same spline in pseudo‐unipolar fashion (intraspline). Figure 1 shows the recording electrode configurations for these modes.

We evaluated the accuracy of the two mapping modes to demonstrate acute PVI at index procedure, comparing these to use of traditional pacing to prove entrance and exit block.

## 2 Methods

In this prospective, single‐centre observational study, EAM was performed after PVI in patients undergoing routine PFA for paroxysmal AF. The procedural workflow, in summary, involved ultrasound‐guided groin access, heparin administered to target an activated clotting time of > 300 s throughout the procedure, quadripolar catheter placement in the coronary sinus, trans‐septal puncture with guidance from fluoroscopy, pressure and iodinated contrast, followed by exchange for the Faradrive sheath. The Farawave Nav ablation catheter was advanced to the left atrium, and ablation of all pulmonary veins was performed with guidance from pre‐procedural computed tomography to demonstrate venous anatomy. This was done with 3 pulses, then rotation for 3 further applications in each of 2 positions (basket and flower) for each upper vein, and 2 pulses in each position in inferior veins as a minimum. Additional lesions were added depending on venous anatomy with the aim of better coverage where necessary of areas such as the anterior rim of the right veins and carina.

After PVI was performed, entrance block was confirmed by moving the Farawave Nav catheter into a small basket position and placing it partly inside each vein. In this position, exit block was then assessed by pacing each spline sequentially. If block had not been achieved, further PFA applications were performed until pacing confirmed block.

Mapping was performed with the Farawave Nav catheter to construct 2 separate maps: one in bipolar “interspline” mode, and a separate map in pseudo‐unipolar, or “intra‐spline” mode. The number of veins isolated according to pacing was then compared to the number of veins displaying as isolated (below voltage threshold of 0.05 mV with absence of signal seen, or independent firing within PVs during CS pacing**)** on the created maps. Individual mapping point signals were inspected and re‐annotated where necessary during the case. Two separate blinded assessors examined each map offline after the case, and where there was disagreement, a third blinded assessor reviewed the maps.

### 2.1 Statistical Analysis

Analysis was performed utilizing R (version 4.3.2, R Foundation for Statistical Computing, Vienna, Austria). Continuous variables are reported as mean ± SD, or median ± IQR for non‐normally distributed parameters and categorical variables are reported as proportions. Wilcoxon rank‐sum tests were used to compare Faraview mapping “interspline” mode with pseudo‐unipolar (“intraspline”) mode. A generalized linear mixed‐effects model with a binomial distribution was fitted to assess the variability in outcomes. The model included Method, Observer, and Patient as random effects to account for clustering within these factors. Variance decomposition was evaluated using intraclass correlation coefficients (ICC) using the performance package. A two‐tailed value of *p* < 0.05 was considered significant.

### 2.2 Patient Consent

Patients were undergoing routine, planned procedures and the new Faraview mapping system was approved for clinical use as an alternative to traditional mapping with other EAM systems. No specific additional processes outside the normal range for this procedure were performed. Further analysis and comparison of maps was performed offline after the procedure and thus no specific additional consent was required, outside standard patient consent for the AF ablation procedure. The project was registered and approved by the ethics committee of the Centre Hospitalier Universitaire (CHU) de Bordeaux. This research was conducted according to the principles of the Declaration of Helsinki.

## 3 Results

Fifteen patients, mean age 58 ± 9 years, 87% male, mean body mass index (BMI) 26.9 ± 4.8 kg/m^2^, Mean left ventricular ejection fraction 60% ± 8%, mean indexed LA volume 24 ± 10 mL had mapping performed with the Faraview system after PVI for paroxysmal AF (See Table 1 for further patient characteristics).

**Table 1:** Baseline characteristics.

Variable	Patients *n* = 15
Age	58 ± 9
Sex = Female	2 (13)
Body Mass Index (kg/m^2^)	26.9 ± 4.8
LVEF (%)	61 ± 8
LA Volume Indexed (mL/m^2^)	24 ± 10
Hypertension	7 (47)
Tye 2 Diabetes Mellitus	1 (7)
Body Mass Index > 27	5 (33)
Obstructive Sleep Apnoea	3 (20)
Continuous Positive Airways Pressure	3 (20)
Alcohol excess	0 (0)
Smoking	4 (27)
Stroke	4 (27)
Ischaemic Heart Disease	0 (0)
Congestive Heart Failure	1 (7)
Myocardial Infarction/PAD/Aortic plaque	1 (7)
Oral anticoagulation	15 (100)
Anti‐hypertensive	6 (40)
Rate control	7 (47)
Anti‐arrhythmic	11 (73)

*Note:* Values shown as mean ± SD or *n* (%).

Abbreviation: PAD, peripheral arterial disease.

### 3.1 Ablation Procedure

All patients had successful PVI as proven by bidirectional pacing for a total of 61 pulmonary veins isolated. A mean of 47 ± 8 PFA applications were delivered for a procedure duration (from perfusion to extubation) of 89 ± 15 min. Table 2 shows further procedural characteristics.

**Table 2:** Procedural characteristics.

Procedural characteristics	
IR	29.23 (6.16)
DAP/PDS (Gy/cm^2^)	5.43 (2.43)
AK (mGy)	53.30 (26.33)
Procedure duration (minutes)	89 (15)
LA Dwell (minutes)	64.20 (11.48)
Mean number of pulsed field ablation lesions	47 (8)
Exit block proven with bidirectional pacing in all pulmonary veins	15 (100)
Bipolar EGMs collected	1205 (708)
Pseudo‐unipolar EGMs collected	1619 (814)
CT LA volume (cc)	146 (28)
Total number of pulmonary veins assessed	61
Number of veins appearing isolated Bipolar mapping mode	
Observer 1	30 (49)
Observer 2	25 (41)
Consensus with observer 3	32 (52)
Number of veins appearing isolated Pseudo‐unipolar mapping mode	
Observer 1	61 (100)
Observer 3	59 (97)
Consensus with observer 3	59 (97)

*Note:* Values shown as mean ± SD or *n* (%).

Abbreviations: CT: computed tomography; DAP, Dose area product; EGM, electrogram; IR, fluoroscopy time; LA, left atrium; PDS Gy, printable dose summary Gray.

### 3.2 Mapping

**Figure 2:**
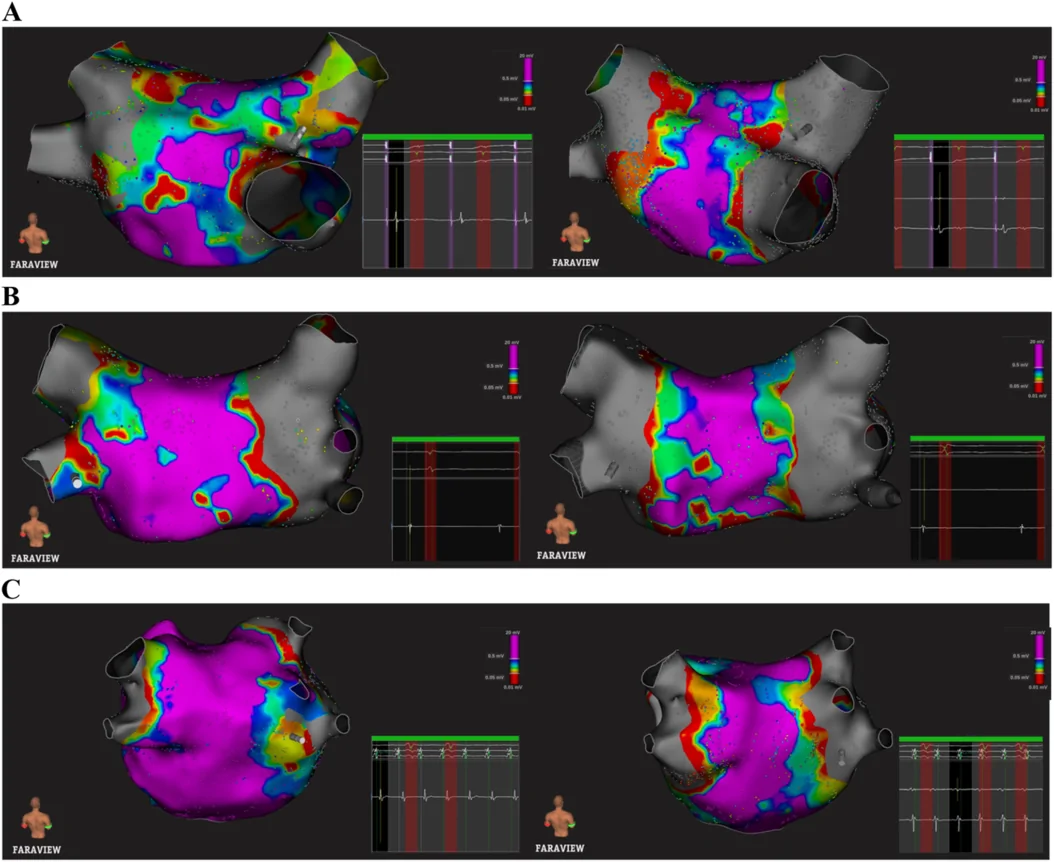
3D maps and signal examples for three patients. The left‐sided panels show bipolar mapping and right‐sided panels show the same patient with pseudo‐unipolar mode. In bipolar mode, (A) appears to show connected right‐sided veins, (B) appears to show left inferior vein connection and (C) appears to show all veins as still connected. The right panels with pseudo‐unipolar mode show all veins are isolated. The roving catheter tip indicates location of individual signals displayed. The left panels show the single reference bipolar electrograms at the mapping points below the ECG reference channels. The right panel recordings show the pseudo‐unipolar electrograms with minimal signal above the lowest electrogram which is the bipolar electrogram at a similar location to those on the left panel maps (maps were taken sequentially, not simultaneously as software does not allow this).

Following PVI, all patients had maps created in each of the 2 mapping modes. Mapping was performed in basket configuration at the PV antrum and flower mode in the LA cavity. Mapping time was 07:52 ± 0:15 min for bipolar mode and 08:40 ± 0:16 min for pseudo‐unipolar mode, with collection of 1175 ± 651 vs. 1662 ± 809 points, respectively. Examples of side‐by‐side maps for patients are shown in Figure 2, with EGMs at selected mapping points showing discrepancy between modes.

### 3.3 Bidirectional Pacing Compared With Each Mapping Mode

On assessment of the 3D EAMs, after consensus between 3 observers, bipolar mapping mode was deemed to show 32/61 (52%) of veins were isolated. With pseudo‐unipolar mapping, consensus was 59/61 (97%) of veins appeared isolated. Individual assessments for each observer are shown in Table 2. Individual points were inspected where necessary for each mapping mode to decide on characteristics of far‐field vs. near‐field signal.

**Figure 3:**
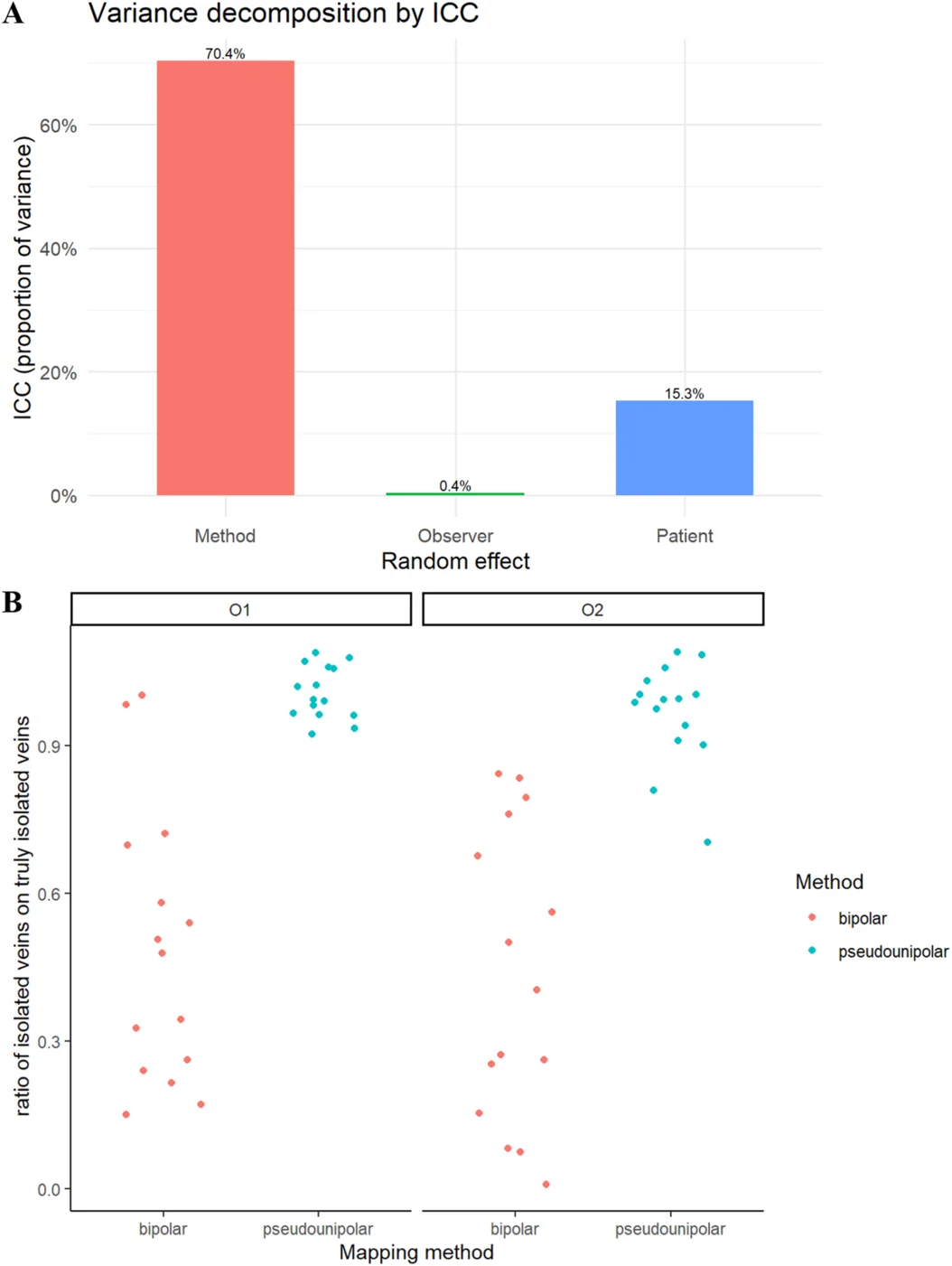
(A) Proportion of variance explained by method, observer and patient. ICC: intraclass correlation. (B) Mapping method and ratio of isolated veins on map to truly isolated veins for each observer.

When testing for cause of variance in assessment, almost all the variance was explained by the mapping method (70.4%), whereas contribution of the observer was very low (0.4%). This indicated that the mapping method had the major influence on results, whereas the effect of observer‐related variability was negligible, and is illustrated in Figure 3A. Figure 3B shows the spread of ratio of isolated veins on mapping to isolated veins by pacing by mapping type and for observer 1 and 2.

## 4 Discussion

This is the first study to compare the recently introduced Faraview mapping modes, utilising the Farawave Nav catheter for mapping with the OPAL HDx EAM system.

Key findings are:

1. Compared with entrance and exit‐block pacing, pseudo‐unipolar mode showed high agreement with assessment of isolation.
2. Interspline mapping did not confirm isolation well and had poor agreement with pacing and pseudo‐unipolar assessment due to increased far‐field signal detection.
3. Pseudo‐unipolar mapping could be performed within 8 min at the end of the procedure.

### 4.1 AF Ablation Development and 3D Mapping Introduction

Traditional radiofrequency ablation for pulmonary vein isolation, which emerged after proof that 90% of AF triggers were from the pulmonary veins did so in the era pre‐EAM [6, 7]. With the introduction of EAM [8], improved catheter localisation ensued, with ongoing improvements in spatial resolution and evolution of ablation lesion metrics improving paroxysmal AF ablation success to > 70% at 12 months [9].

### 4.2 Non‐Mapping Approaches to AF Ablation

Cryoablation was introduced as a single‐shot alternative to RF, and was shown to have equivalent efficacy [10]. This procedure was initially introduced with fluoroscopy guidance only and optional work‐arounds to augment with the use of mapping have been implemented by some operators [11].

Pulsed Field ablation was introduced in 2019 with initial data on remapping studies showing incredible efficacy at close to 100% at 3 months [2]. This technology was also introduced as fluoroscopy guided.

Unfortunately, these initial results have not been confirmed in subsequent multi‐centre randomised controlled trials, with efficacy shown to be similar to traditional thermal methods at 70%–80% success at 12 months [1, 12, 13].

### 4.3 Mapping Addition to PFA

In the recent MANIFEST‐REDO registry, patients with AF recurrence post PVI performed with PFA were shown to have pulmonary vein reconnection in 55% of cases [3]. Importantly, there was an association between reduced reconnection rate and the use of 3D EAM at the index procedure. This is despite the known limitations of mapping post PFA, due to inability to assess whether ablation lesions are durable, or might contain regions of reversibility giving tissue stunning but reconducting after recovery from this phenomenon. This and other data suggest that improved durability of PVI should be obtained with the use of EAM [14].

Advantages of the Faraview approach are the ability to use the pentaspline Farawave Nav ablation catheter for both mapping and ablation, thus avoiding the need for the use and cost of a separate high‐density mapping catheter, and avoiding the need for catheter exchanges. The ability to map more easily during the workflow may reduce the need to deliver additional “insurance” lesions, or conversely direct additional lesions to areas not otherwise appreciated to have poor lesion coverage. The disadvantages of the Faraview approach included wider electrode spacing than traditional high‐density mapping catheters given it was not originally designed for this function, and the derived nature of the virtual unipoles which means fewer collected points per catheter position. The geometry around PV orifices also involves some assumptions about catheter shape and orientation which lead to requirement for significant editing in real‐time to obtain a closer resemblance to the true anatomy as shown on CT, though this can be significantly improved by aiming for a coaxial position when mapping these structures. As this was a population with paroxysmal AF and generally healthy LA voltage, this may have increased the likelihood of far‐field signal detection with the interspline mapping mode. The 2 veins in the “intraspline” mapping group with observer concordance discrepancy with isolation shown on pacing were one RIPV and one LIPV where signal seen may have been consistent with superior vena cava and left atrial appendage far‐field signal, respectively, but was deemed more extensive than expected for these veins. Exit block was proven for all PVs. The results of this study suggest that if mapping is required, pseudo‐unipolar mode should be chosen given its increased accuracy.

## 5 Limitations

The small sample size and single‐centre nature of this study are limitations. Nevertheless, the difference between mapping modes in comparison to pacing assessment was marked, which enables meaningful comparison. Despite mapping being enabled, tissue contact indicators remain unavailable such as contact force, temperature, and impedance changes during ablation. Intracardiac echocardiography (ICE) is a modality that could improve assessment of tissue contact and catheter orientation, but this is not part of routine workflow, partly due to cost. Full mapping was performed post‐ablation rather than during ablation, with lesion sets projected onto the map. This may or may not be more important to more readily guide catheter position. The effect of mapping on outcomes is not studied here, which would require randomisation and a larger study to assess.

## 6 Conclusions

When performed post‐ablation, the recently‐introduced pseudo‐unipolar Faraview Nav EAM mode shows good agreement with traditional pacing methods of assessing pulmonary vein isolation. This mode appears the more accurate for assessment post‐PVI compared with bipolar inter‐spline mode using the same system. The study methods and results are summarised in Central Illustration 1.

**Central Illustration 1:**
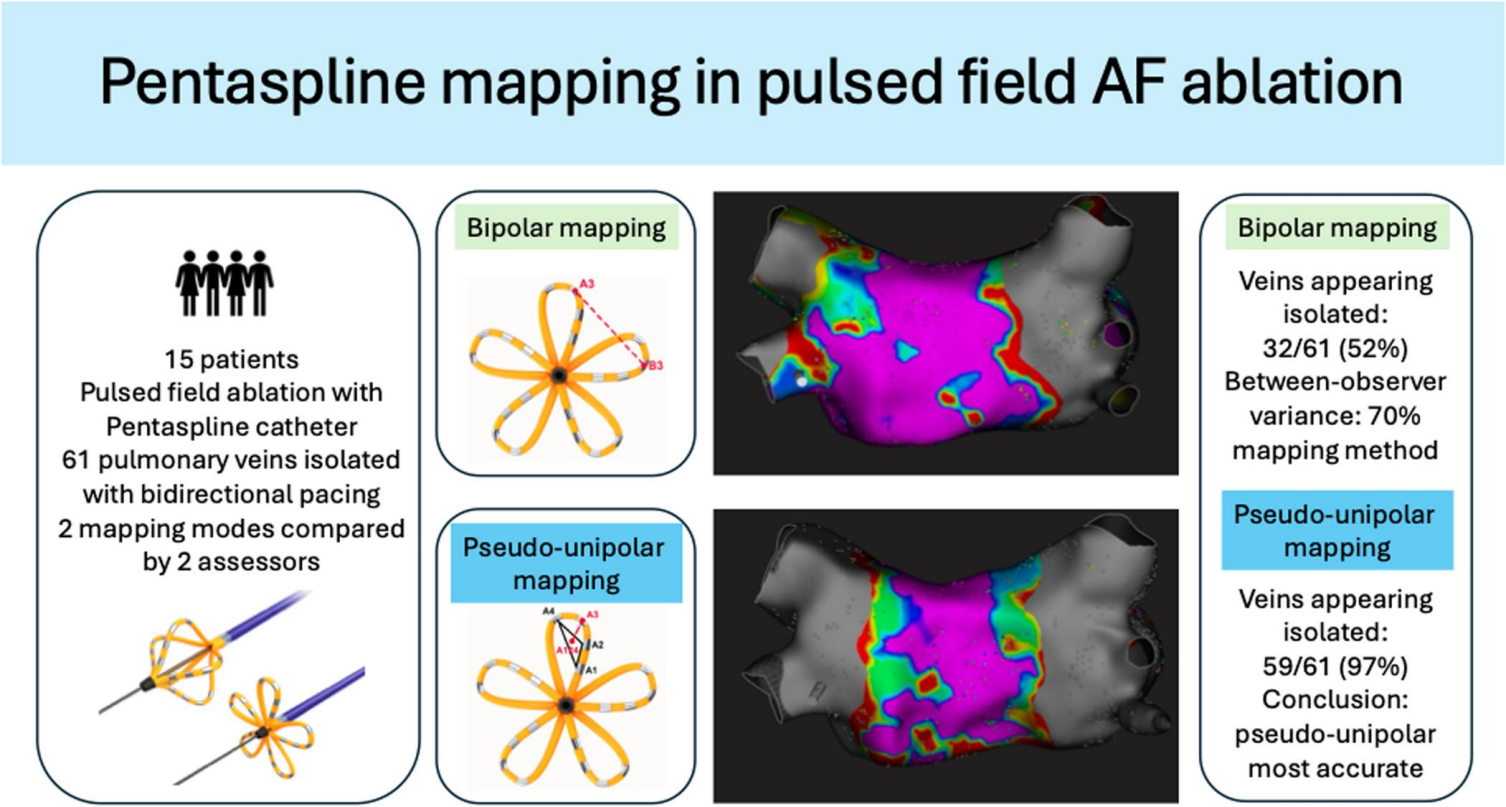
Pentaspline mapping in pulsed field AF ablation.

## Disclosure

Dr. Fitzgerald was supported by a postgraduate scholarship and research grant from the University of Adelaide. All other authors have reported that they have no relationships relevant to the contents of this paper to disclose.

## Conflicts of Interest

The authors declare no conflicts of interest.

## Data Availability

The data that support the findings of this study are available on request from the corresponding author. The data are not publicly available due to privacy or ethical restrictions.
